# Antibiotics-encapsulated nanoparticles as an antimicrobial agent in the treatment of wound infection

**DOI:** 10.3389/fimmu.2024.1435151

**Published:** 2024-10-29

**Authors:** Mohammad Taheri, Mohammad Reza Arabestani, Fereshte Kalhori, Sara Soleimani Asl, Masoumeh Asgari, Seyed Mostafa Hosseini

**Affiliations:** ^1^ Department of Microbiology, School of Medicine, Hamadan University of Medical Sciences, Hamadan, Iran; ^2^ Department of Anatomical Sciences, School of Medicine, Hamadan University of Medical Sciences, Hamadan, Iran; ^3^ Department of Nutritional Sciences, School of Medicine, Hamadan University of Medical Sciences, Hamadan, Iran; ^4^ Nutrition Health Research Center, Hamadan University of Medical Sciences, Hamadan, Iran

**Keywords:** wound healing, ciprofloxacin, vancomycin, solid lipid nanoparticles, *Pseudomonas aeruginosa*, *Staphylococcus aureus*

## Abstract

Disruption in the wound-healing process is caused by the presence of bacteria and leads to major problems and delays in wound healing. The limitations of commonly used medicines for treating wound infections include drug toxicity, insufficient microbial coverage, poor penetration, and increased resistance. This study aimed to determine the effect of ciprofloxacin loaded in solid lipid nanoparticles (Cip-SLN) on *Pseudomonas aeruginosa* and ampiciliin-vancomycin loaded in solid lipid nanoparticles (Amp-Van-SLN) on *Staphylococcus aureus* in wounds. Antibiotics were encapsulated in SLNs using the double emulsion method and were characterized. The *in-vitro* effect of antibiotic-loaded nanoparticles on *P. aeruginosa* and *S. aureus* was assessed using well diffusion and MIC methods. Finally, the topical antibacterial activity of these nanoparticles against bacterial wound infection was measured in a mouse model. MIC results showed that in the first 24 hours, the free drug had a greater effect on inhibiting bacteria, and in 72 hours, the inhibitory effect of nanoparticles increased. There was no toxicity effect of 400 µg/mL of nanoparticles on cells. According to the findings, the groups treated with Cip-SLN and Amp-Van-SLN were more effective than the control group (untreated) in different concentrations. In the wound healing process, the group treated with solid lipid nanoparticles (SLNs) exhibited a greater epithelial thickness, indicating enhanced healing, compared to the group treated with the free drug. The use of SLN can increase the accumulation of antibiotics at the site of infection with a slow release of the drug due to its fatty nature, which leads to a significant inhibitory effect on bacteria and also improves wound healing.

## 1 Introduction

The skin serves as a vital barrier against microorganisms and pathogens, but when wounds occur, the risk of infection increases as normal flora and pathogens can enter the bloodstream (1). The medical field aims to expedite wound healing with fewer side effects to reduce infections, which remain a significant risk factor for disease and mortality despite advancements (2). Burn wounds, causing approximately 265,000 annual deaths worldwide, particularly affect low- and middle-income countries, often necessitating surgery and antimicrobial treatment for severe cases (3). Conversely, chronic ulcers are frequently linked to underlying conditions like diabetes or immunodeficiency, impairing the immune system’s ability to protect against infections which may lead to organ failure and death (4). Overall, approximately 10-20% of wounds become infected, posing considerable health risks, delaying healing, and increasing antibiotic usage, and treatment costs (5). The emergence of Methicillin-resistant *S. aureus* (MRSA) strains in response to methicillin during the 1960s raised concerns, forcing the adoption of vancomycin as the primary treatment. However, the rise of vancomycin-resistant *S. aureus* strains due to limited alternative treatment options became a global issue (6, 7). *P. aeruginosa*, found commonly in chronic wounds, poses challenges due to its resistance to various antibiotics, particularly in the presence of biofilms, hindering wound healing and leading to chronic conditions (8). Bacterial biofilms, containing pathogenic bacteria encased in an exopolysaccharide layer, exhibit increased resistance to immune responses and conventional antibiotics, complicating treatment (9). A biofilm structure contains pathogenic bacteria encapsulated in an exopolysaccharide layer, which communicate with each other through the secretion of signaling molecules that differentiate physiology, phenotype, and expression of altered genes compared to planktonic cells. Bacteria living in a biofilm are generally resistant to host immune responses and treatment (10). To enhance drug efficacy in wound management, Solid lipid nanoparticles (SLNs) have emerged as a promising drug delivery system to improve bioavailability and treatment efficiency (11). Nanoparticles, including SLNs, have shown potential in enhancing the effectiveness of antibiotics, particularly for wound healing applications when combined with antimicrobial (12). Nanocarriers carrying antibiotics have emerged as a promising option in drug delivery (13). Studies have shown that developing novel pharmaceuticals alone is ineffective due to the poor water solubility of certain drugs and the low viability of new drug molecules. Hence, focusing on developing drug delivery systems that can address these challenges is crucial. These carrier systems should be non-toxic, can encapsulate a sufficient amount of drug, and enable targeted and controlled drug release. Solid lipid nanoparticles (SLNs) have been investigated as a carrier system for various applications in recent studies. One of the key advantages of SLNs is that they are composed of natural, non-toxic, non-immunogenic, and biodegradable lipid molecules (14). The effectiveness of SLN loaded with antibiotics was evaluated *in vitro* and *in vivo* conditions to improve efficiency and minimize the use of conventional antibiotics in the treatment of bacterial wound infections. The study aimed to evaluate whether encapsulating antibiotics in solid lipid nanoparticles could improve their efficacy against wound infections by enhancing drug delivery, prolonging drug release, and reducing bacterial colonization in a mouse model.

## 2 Methods

### 2.1 Synthesis and evaluation of nanoparticles

The nanoparticle was synthesized by the double emulsion/melt dispersion technique. To find the optimal formulation, different percentages of lipid, surfactant, and antibiotic concentrations were investigated. Stearic acid was first heated to 70°C (5°C higher than the melting point), then pre-dissolved and warmed poloxamer 407/lecithin, and the antibiotics were added to the molten lipid, and magnetic stirring was used (150 rpm). After that, the mixture was homogenized with warm distilled water and the mixture was sonicated for 60 seconds at 45°C. In the second phase, heated tween-80 was added to the initial emulsion, and the mixture was homogenized by an ultrasonic device at 45°C for 2 minutes to obtain the second emulsion (15 seconds on, 5 seconds off). To stabilize the produced nanoparticles, the resultant mixture was gently added to cold distilled water (5°C) and disseminated in the solution for 5 minutes. For the preparation of free SLN, the same circumstances were observed (drug-free solid lipid nanoparticles). Finally, to separate the free drug, the nanoparticles were washed three times with distilled water using a high-speed centrifuge (25,000 rpm for 15 minutes). Almost 500 mg of nanoparticles was added to 1 mL glycerin and lyophilized at -80°C using a vacuum pump (lyophilizer). The nanoparticles were first dissolved in distilled water and sanitized with a 450 nm filter before being used in biological experiments (15).

### 2.2 Nanoparticle characterization and cytotoxicity assay

Mean particle size, PDI and zeta potential of nanoparticles were measured. The maximum wavelength (λ Max) of antibiotics was determined and diluted and the standard curve of the drug was plotted. The amount of loading and encapsulation of antibiotics in the synthesized nanoparticles was determined using a spectrophotometer (an indirect method). Briefly, 10 mg of nanoparticles were added to 10 mL of distilled water. Next, the resulting suspension was centrifuged at 1500 rpm for 15 minutes. The supernatant was analyzed using a spectrophotometer (2100 UV, USA) at the optimum wavelength for each antibiotic. The thermal behavior of optimal formulation and its components were evaluated by differential scanning calorimetry (DSC) (METTLER TOLEDO-DSC 1). For the spectroscopic examination of the samples, the synthesized lyophilized NP (optimal formulation) was mixed with some potassium bromide (KBr) simultaneously and converted into compact discs by a hydraulic compressor. The disks were then recorded in the path of infrared light in the middle IR range (400-400 cm-1) using an FTIR spectroscope (PerkinElmer, spectrum 400, America). Drug release from the synthesized nanoparticle was performed using the dialysis bag method (cut-off 12-14 kDa). Drug release was quantified by measuring the concentration of the released antibiotic in the surrounding solution at regular time intervals using a UV-visible spectrophotometer at the antibiotic’s maximum absorbance wavelength.

The stability of the optimal formulation in suspension was evaluated for the short-term (one week in terms of appearance and one month in terms of appearance and physicochemical properties) and long-term (6 to 12 months in terms of appearance and physicochemical properties). The emission scanning electron microscope (Fe-SEM) was used to examine the morphology of the nanoparticle. To evaluate the cytotoxicity of the synthesized nanoparticles and according to the instructions of the kit (Kiazist Kit/Iran), the human epidermoid carcinoma epithelial cell line (A431) was used. The dilutions studied were 25, 50, 100, 200 and 400, 800 µg of nanoparticles and free drugs. Cell viability was assessed using the MTT assay at 570 nm. The basis of comparisons was 100% survival of control samples.

### 2.3 Well diffusion and MIC

According to CLSI, recommended well diffusion and MIC methods for studied nanoparticle efficacy were used. For well diffusion assay, the bacterial suspension was diluted to yield 10^4^ CFU/mL, which was then employed in the antibacterial studies. Using a sterile cotton swab, a lawn of bacterial culture prepared above was spread uniformly on the Muller–Hinton agar plates. Using a cylindrical glass tube were punched into the bacteria-coated Muller–Hinton agar plates. 100 µL of NP sample solution were added to each well and were incubated at 37°C for 18 h. The inhibition zone diameter which reflects the susceptibility of bacteria to the NPs, was measured (16).

According to the CLSI recommendations, the minimum inhibitory concentration (MIC) was established using the microdilution technique. To summarize, all strains were cultivated on Brain Heart Infusion agar, and three or four colonies were floated in fresh sterile saline solution to achieve an initial concentration of 0.5 McFarland (1.5 10^8^ CFU/mL). 100 µL of the 1:100 diluted cell suspensions were dispensed in each well. The MICs were established as the lowest dilution of nanoparticles capable of inhibiting observable bacterial growth after a 24-hour incubation period at 37°C (16).

### 2.4 Wound formation and treatment

In this study, male rats aged 6 to 8 weeks were utilized to create surgical wounds, with each group consisting of five rats. The rats with infectious wounds caused by *P. aeruginosa* were treated with various compounds, including; 1) Cip-SLN 2) free ciprofloxacin, 3) free SLN and 4) positive control (Wounded rats were untreated) and Rats with infectious wounds by Staphylococcus strains were treated with 1) Van-Amp-SLN, 2) Free Van-Amp, 3) Van-SLN. 4) Amp-SLN 6) free SLN and 7) positive control (Wounded rats were untreated) Ketamine (50 mg/g body weight) and xylazine (5 mg/g body weight) were injected intraperitoneally for anesthesia. The back of earea of each male rat’s neck was disinfected and the hair in this region was shaved in a one-centimeter circular area. Subsequently, a one-centimeter incision was made to create a wound at this site, and 1.5×10^6^ of *P. aeruginosa* bacteria were inoculated into the wound. The survival of each rat was monitored daily. Upon detection of infection in the wounds, sterile adhesive dressings were applied half an hour after administering the drug treatment. Samples were collected using sterile swabs every two days to assess the bacterial load. The wound healing process and sample collection continued for two weeks following the identification of wound infection. Animal studies were performed following the ARRIVE guidelines. The ethics committee of Hamadan University of Medical Sciences gave its approval for this study, by the US National Institutes of Health guidelines.

The CFU counting method was used to assess the number of live *P. aeruginosa* and *S. aureus* bacteria in wound samples for up to 14 days (every other day). Following the observation of the infection, a sterile swab was used to collect the samples, which were then transferred to a flask containing 1 mL of sterile normal saline. Different dilutions were prepared from the sample suspension and then 100 μl of each dilution was cultured on a chocolate agar plate for 24 hours in an incubator at 37°C. The colonies’ units were then counted and the findings were reported as (CFU/mL) (17).

### 2.5 Histopathology

The macroscopic examinations were carried out by watching the wound’s appearance and the daily healing process. All wound samples were isolated and immersed in 10% formalin for 24 hours. Light microscopy was used to analyze skin morphology, and collagen synthesis after a 5-micron diameter incision was made with a microtome device and hematoxylin-eosin (18).

### 2.6 Ethical statement

The study was conducted under the ethics approval code IR.UMSHA.REC.1400.770.

### 2.7 Statistical analysis

The data is presented as a mean deviation with three repetitions. Data were analyzed using SPSS software version 20 and one-way ANOVA. A comparison of microbial load in different treatment groups was performed using the Tukey test. P <0.05 was considered significant.

## 3 Results

### 3.1 Properties of nanoparticles

**Figure 1:**
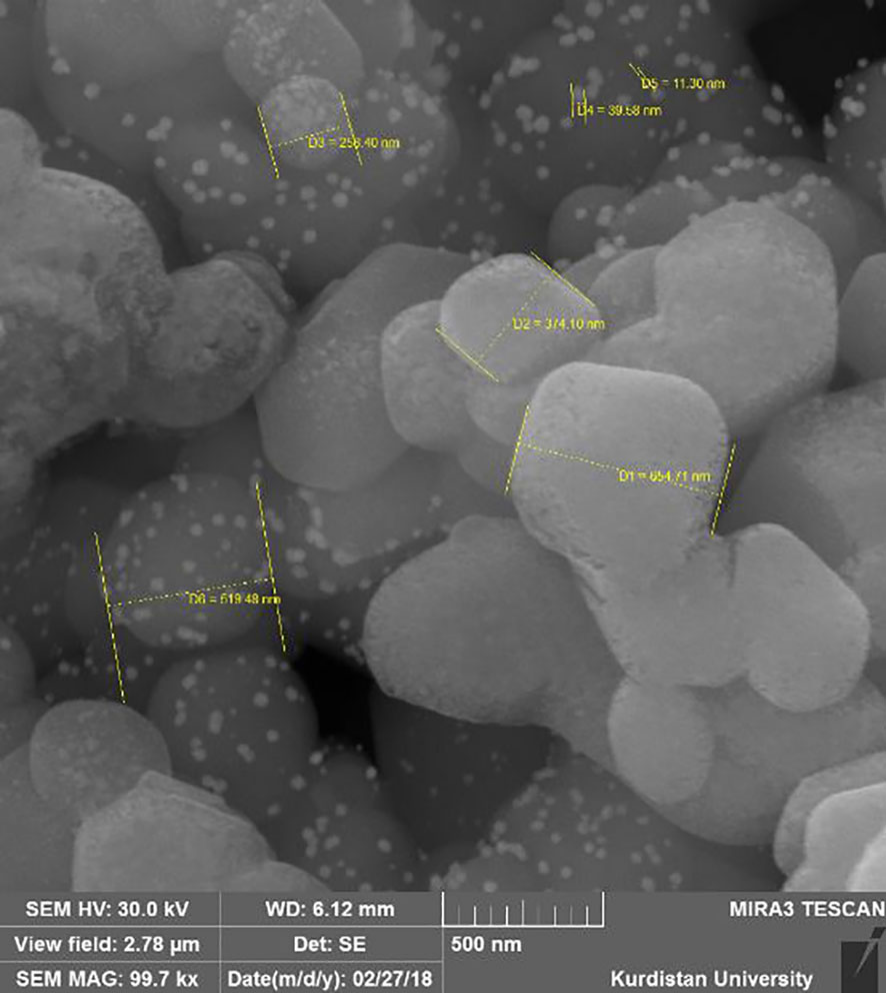
Field emission scanning electronic microscope images of NPs.

The average size, zeta potential, and PDI of Cip-SLN were (300 ± 25 nm), (−18.5 ± 2.5 mV) and (0.295 ± 0.032), respectively. The amount of loading and encapsulation of ciprofloxacin in the synthesized nanoparticle was 18.1 ± 1.4% and 96.8 ± 3.2%, respectively. Particle size, PDI, zeta potential, drugs loading, and encapsulation efficiency of Amp-Van-SLN were 290 ± 31 nm, 0.350 ± 0.022, −17.9 ± 2.3 mV, 15.7 ± 1.5%, and 95.15 ± 2.6%, respectively. The results of the stability test showed that the size of the nanoparticles did not change much until the ninth month, but after 12 months, the size of the nanoparticles showed an increase of 11.5% (**Table 1**). The results of morphology analysis with Fe-SEM showed that the particles were spherical and had a smooth surface with homogeneous dispersion, as shown in **Figure 1**, the synthesized nanoparticle has a size of about 250 to 400 nm according to the scale bar.

**Table 1:** Technological characteristics of NPs: average diameter, Polydispersity Index (PDI) and zeta potential (ZP) throughout stability study, (means ± SD, n = 3).

Formulation	Technological parameters	Time (months)					
		0	2	4	6	8	12
Cip-SLN	Size (nm)	300 ± 25	305 ± 27	309 ± 25	312 ± 25	324.5 ± 11	334.5 ± 31*
	PDI	0.295 ± 0.032	0.302 ± 0.022	0.294 ± 0.031	0.315 ± 0.012	0.315 ± 0.19	0.324 ± 0.12
	ZP (mV)	−18.5 ± 2.5	−16.1 ± 2.2	−17.5 ± 1.5	−15.5 ± 2.1	-14.9 ± 3.1	-15.4 ± 3.2
Amp-Van-SLN	Size (nm)	290 ± 31	295 ± 23	289 ± 33	302 ± 21	314.8 ± 31	324.8 ± 24*
	PDI	0.350 ± 0.022	0.357 ± 0.012	0.321 ± 0.012	0.341 ± 0.022	0.321 ± 0.012	0.338 ± 0.014
	ZP (mV)	−17.9 ± 2.3	−16.4 ± 3.1	−15.9 ± 2.1	−16.9 ± 2.1	-15.25 ± 1.4	-14.25 ± 1.9

*p value < 0.05.

**Figure 2:**
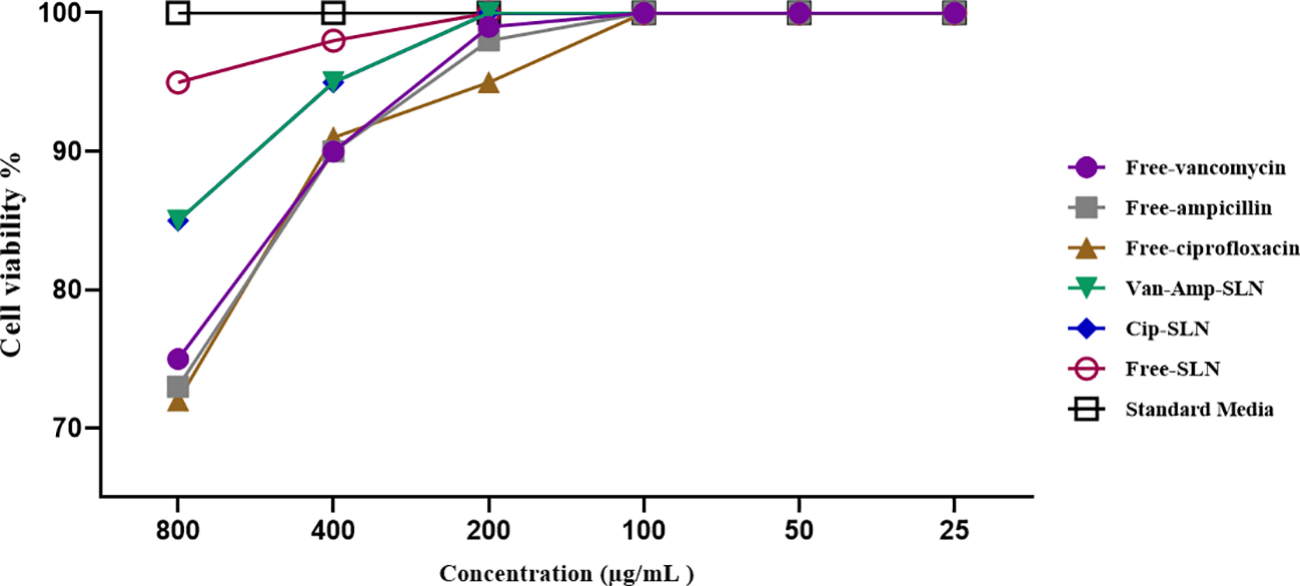
NPs and free drugs *in vitro* release profile from the SLN formulation in pH = 7.4 phosphate buffer (n = 3).

The results of FTIR and DSC showed that in the nanoparticle synthesis process, there was no chemical reaction that would create a new chemical compound, and drugs were placed in a molecular form in the lipid matrix and not in a crystalline and free form. In terms of the release time, the results showed that 82 hours are needed for 80% of drugs to be released from the nanoparticle (**Figure 2**).

### 3.2 MIC and well diffusion results

According to agar well diffusion method data, free drugs had a greater effect on bacteria than nanoparticles. But, after 72 hours of incubation, the inhibition zone diameter of nanoparticles was increased gradually showing the slow release rate of nanoparticles (**Tables 2**, **3**).

**Table 2:** Results of well diffusion test after *Pseudomonas aeruginosa* treatment with free ciprofloxacin and Cip-SLN.

Antibacterial activity								
Zone of inhibition (mm) in three times (h) and concentration(μg/mL)								
Free Ciprofloxacin								
24 h			48 h			72 h		
0.25	0.5	1	0.25	0.5	1	0.25	0.5	1
19	27	30	20	28	34	20	28	34
Ciprofloxacin -SLN								
24 h			48 h			72 h		
0.25*	0.5	1	0.25	0.5	1	0.25	0.5	1
11	11	13	13	14	17	18	19	27

*Based on loading ratio.

**Table 3:** Results of well diffusion and MIC test after *Staphylococcus strains* treatment with free drugs and NPs.

Bacterial strain	Antibacterial activity																										
MRSA	Zone of inhibition (mm) in three time (24, 48 and 72h) and three concentration (100-50-25 μg/mL) and (2.5-1.25-0.625 μg/mL)																										
	Free vancomycin (2.5-1.25-0.625 μg/mL)									Free ampicillin (100-50-25 μg/mL)									Free vancomycin & ampicillin (2.5-1.25-0.625 & 100-50-25 μg/mL)								
	24h			48h			72h			24h			48h			72h			24h			48h			72h		
	25	22	19	25	21	19	25	22	18	21	18	15	20	18	16	21	19	16	25	23	20	25	22	21	25	22	20
	Van-SLN (2.5*-1.25-0.625 μg/mL)									Amp-SLN (100-50-25 μg/mL)									Van-Amp-SLN (2.5-1.25-0.625 & 100-50-25 μg/mL)								
	24h			48h			72h			24h			48h			72h			24h			48h			72h		
	15	13	12	19	17	15	24	20	16	20	17	15	23	19	18	26	21	19	20	18	15	22	19	17	29	25	22
MSSA	Free vancomycin (2.5-1.25-0.625 μg/mL)									Free ampicillin (100-50-25 μg/mL)									Free vancomycin & ampicillin (2.5-1.25-0.625 & 100-50-25 μg/mL)								
	24h			48h			72h			24h			48h			72h			24h			48h			72h		
	30	28	27	30	29	27	30	28	28	31	30	25	32	30	25	31	31	26	32	31	29	32	32	29	33	31	29
	Van-SLN (2.5-1.25-0.625 μg/mL)									Amp-SLN (100-50-25 μg/mL)									Van-Amp-SLN (2.5-1.25-0.625 & 100-50-25 μg/mL)								
	24h			48h			72h			24h			48h			72h			24h			48h			72h		
	14	13	11	18	15	13	30	24	19	19	16	14	19	17	15	32	27	21	19	16	15	27	23	20	34	29	27
VISA	Free vancomycin (2.5-1.25-0.625 μg/mL)									Free ampicillin (100-50-25 μg/mL)									Free vancomycin & ampicillin (2.5-1.25-0.625 & 100-50-25 μg/mL)								
	24h			48h			72h			24h			48h			72h			24h			48h			72h		
	15	10	9	15	10	10	15	11	10	17	15	14	17	15	15	17	15	15	17	14	13	17	15	13	17	15	13
	Van-SLN (2.5-1.25-0.625 μg/mL)									Amp-SLN (100-50-25 μg/mL)									Van-Amp-SLN (2.5-1.25-0.625 & 100-50-25 μg/mL)								
	24h			48h			72h			24h			48h			72h			24h			48h			72h		
	8	5	0	11	7	2	13	9	4	15	12	10	18	15	12	25	20	18	16	11	10	20	15	13	27	20	18

*Based on loading ratio.

MSSA, methicillin-sensitive *Staphylococcus aureus*; MRSA, Methicillin-resistant *Staphylococcus aureus*; VISA, Vancomycin-intermediate *Staphylococcus aureus*.

**Figure 3:**
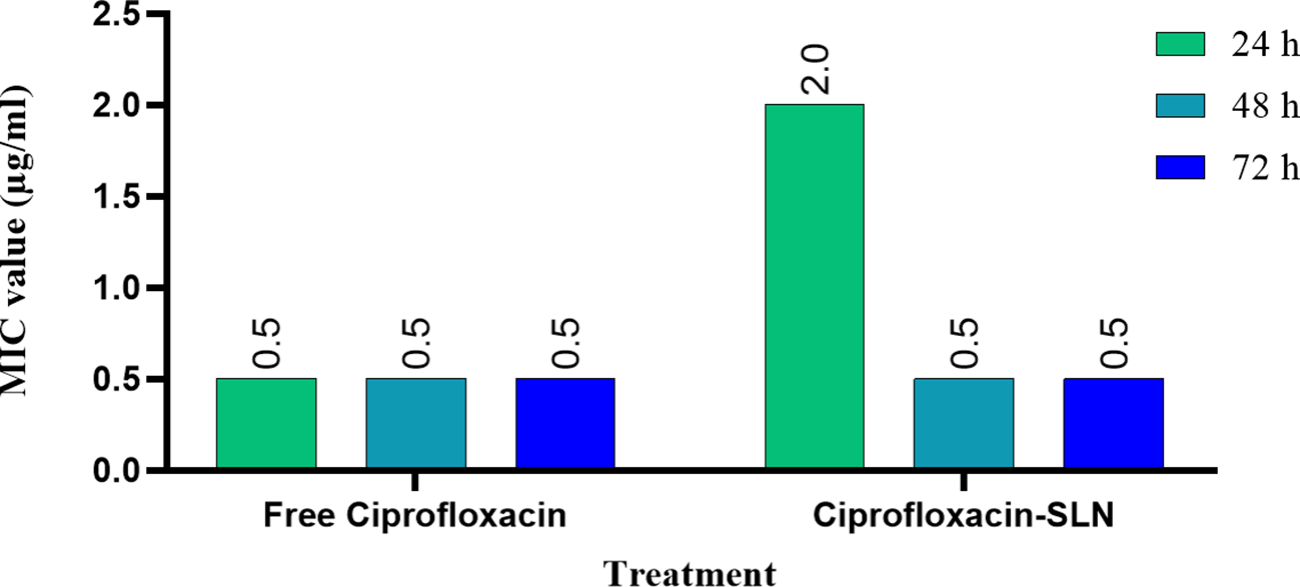
Results of MIC test after treatment with free ciprofloxacin and Cip-SLN.

**Figure 4:**
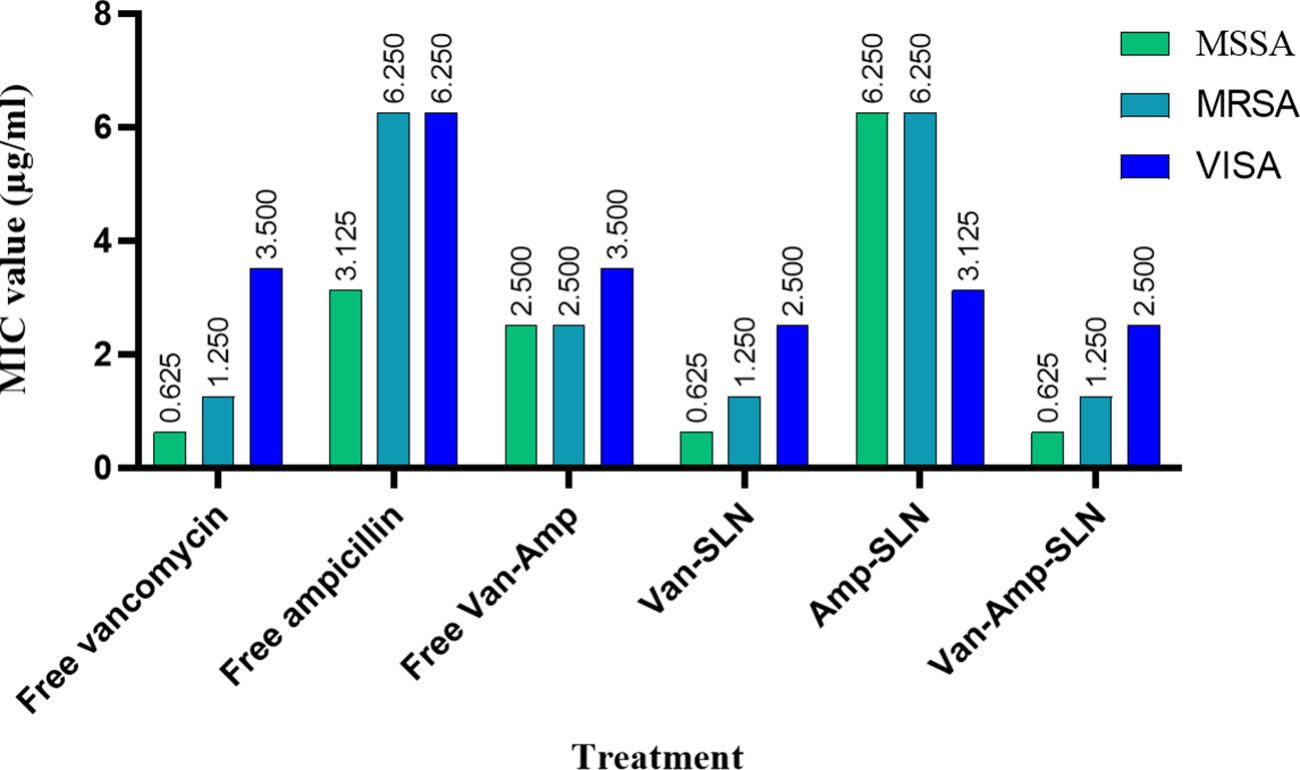
Results of MIC test for staphylococcus strains after treatment with free drugs and NPs.

The results of MIC showed that in the first 24 hours, the free drug had a better effect on the bacteria by decreasing the mic, but it was equal to the passage time of the mic of the free drug and nanoparticle, and in 72 hours, the mic of the free drug increased (**Figures 3**, **4**).

### 3.3 Cytotoxicity assay using MTT

**Figure 5:**
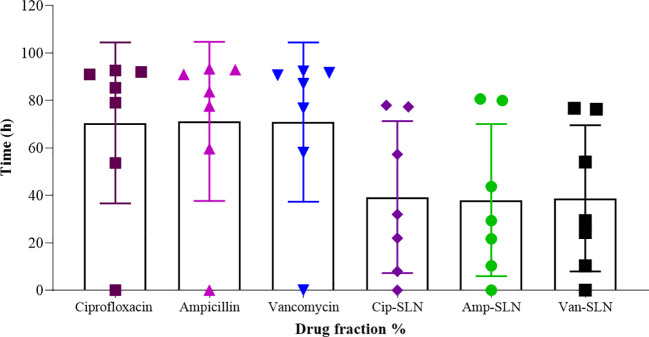
The effect of free drugs and NPs on fibroblast cell lines, the vertical axis shows the percentage of living cells and the horizontal axis shows the concentration of different formulations.

Cytotoxicity investigation of different concentrations of free ciprofloxacin, free vancomycin, free ampicillin, Cip-SLN, Amp-Van-SLN, and free SLN on human epidermoid carcinoma epithelial cell line (A431) indicated that there was no toxic effect of the drugs and nanoparticles up to a concentration of 200 µg/mL (**Figure 5**).

### 3.4 Antimicrobial test results

**Figure 6:**
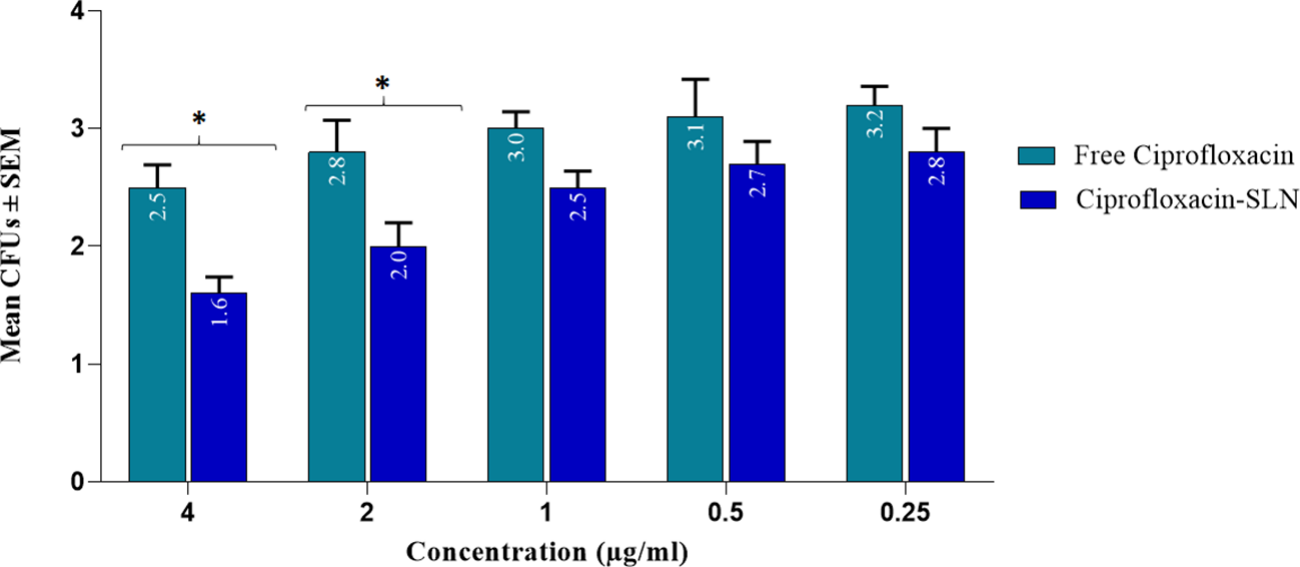
Bacterial counts (CFU Log10) of rat skin at 0, 4, 9, and 14 days after treatment with free ciprofloxacin and Cip-SLN, the vertical axis shows the average bacterial colony count (CFUs) and the horizontal axis shows the concentration value of free ciprofloxacin and ciprofloxacin-SLN. *p-value < 0.05.

The effect of different formulations on reducing the number of *S.aureus* colonies in infectious wounds of different groups of rats is shown in **Table 4**. According to the results, the most efficacy was related to Amp-Van-SLN which significantly reduced 66% of the *S.aureus* colony count at the site of infection (P <0.05). Comparing the treated groups, it was found that nanoparticles containing two antibiotics have a better effect than free antibiotics and have significantly reduced the number of bacteria at the site of infection. The effect of Cip-SLN on reducing the number of *P.aeruginosa* colonies in infected wounds of rats in different groups is shown in **Figure 6**. According to the results, Cip-SLN was more effective than the control group (infection without treatment) in different concentrations, which caused a 64% reduction in the number of bacteria at the site of infection, which was remarkably significant (p<0.05). Also, Cip-SLN is more effective than free drugs and leads to a bacterial burden reduction at the site of infection.

**Table 4:** Bacterial counts (CFU Log10) of rat skin at 4, 9, and 14 days before and after treatment with free drugs and NPs.

Bacterial strain	day	Free-Van		Free-Amp		Free-Van & Amp		Van-SLN		Amp-SLN		Van-Amp-SLN		Untreated
		CFU (Log_10_)	Log CFUs reduction	CFU (Log_10_)	Log CFUs reduction	CFU (Log_10_)	Log CFUs reduction	CFU (Log_10_)	Log CFUs reduction	CFU (Log_10_)	Log CFUs reduction	CFU (Log_10_)	Log CFUs reduction	CFU (Log_10_)
MRSA	0	6.5 ± 0.03	0.0	6.6 ± 0.01	0.0	6.5 ± 0.02	0.0	6.5 ± 0.01	0.0	6.5 ± 0.03	0.0	6.5 ± 0.03	0.0	6.6 ± 0.01
	4	6.4 ± 0.01	0.1	6.3 ± 0.02	0.2	6.3 ± 0.01	0.2	6.0 ± 0.01	0.5	6.4 ± 0.01	0.1	6.0 ± 0.01	0.5	6.5 ± 0.03
	9	4.5 ± 0.02	0.0	4.4 ± 0.01	0.1	4.2 ± 0.02	0.3	3.3 ± 0.03	0.8	3.5 ± 0.01	1.0	2.7 ± 0.01	1.8	4.5 ± 0.04
	14	3.1 ± 0.03	0.2	4.0 ± 0.04	0.3	3.8 ± 0.03	0.5	2.5 ± 0.02	0.8	2.7 ± 0.01	1.0	1.5 ± 0.01	1.8	3.3 ± 0.03
MSSA	0	6.7 ± 0.04	0.0	6.7 ± 0.01	0.0	6.7 ± 0.01	0.0	6.6 ± 0.09	0.0	6.7 ± 0.04	0.0	6.7 ± 0.04	0.0	6.7 ± 0.01
	4	6.5 ± 0.02	0.1	6.4 ± 0.03	0.2	6.1 ± 0.03	0.5	6.3 ± 0.01	0.3	6.0 ± 0.02	0.6	5.5 ± 0.01	1.1	6.6 ± 0.02
	9	3.5 ± 0.01	0.6	3.7 ± 0.02	0.4	3.2 ± 0.02	0.9	3.1 ± 0.02	1.0	3.0 ± 0.01	1.1	2.5 ± 0.01	1.6	4.1 ± 0.01
	14	2.1 ± 0.03	1.1	2.3 ± 0.01	0.9	2.0 ± 0.04	1.2	2.0 ± 0.01	1.2	1.7 ± 0.03	1.5	1.3 ± 0.04	1.9**	3.2 ± 0.02
VISA	0	6.0 ± 0.01	0.0	6.0 ± 0.01	0.0	6.0 ± 0.01	0.0	6.0 ± 0.01	0.0	6.0 ± 0.01	0.0	6.0 ± 0.01	0.0	6.0 ± 0.01
	4	5.5 ± 0.03	0.2	5.4 ± 0.03	0.3	5.4 ± 0.01	0.3	4.5 ± 0.01	1.2	4.4 ± 0.02	1.1	4.1 ± 0.01	1.6	5.7 ± 0.03
	9	2.9 ± 0.02	1.0	3 ± 0.01	0.9	2.8 ± 0.03	1.1	2.2 ± 0.03	1.7*	2.1 ± 0.06	1.8*	2.0 ± 0.02	1.9**	3.9 ± 0.02
	14	2.0 ± 0.03	1.0	2.1 ± 0.02	0.9	2.1 ± 0.03	0.9	1.5 ± 0.03	1.5	1.2 ± 0.03	1.8*	1 ± 0.01	2.0**	3.0 ± 0.03

*P-value < 0.05. **P-value ≤ 0.01.

### 3.5 Wound healing

**Figure 7:**
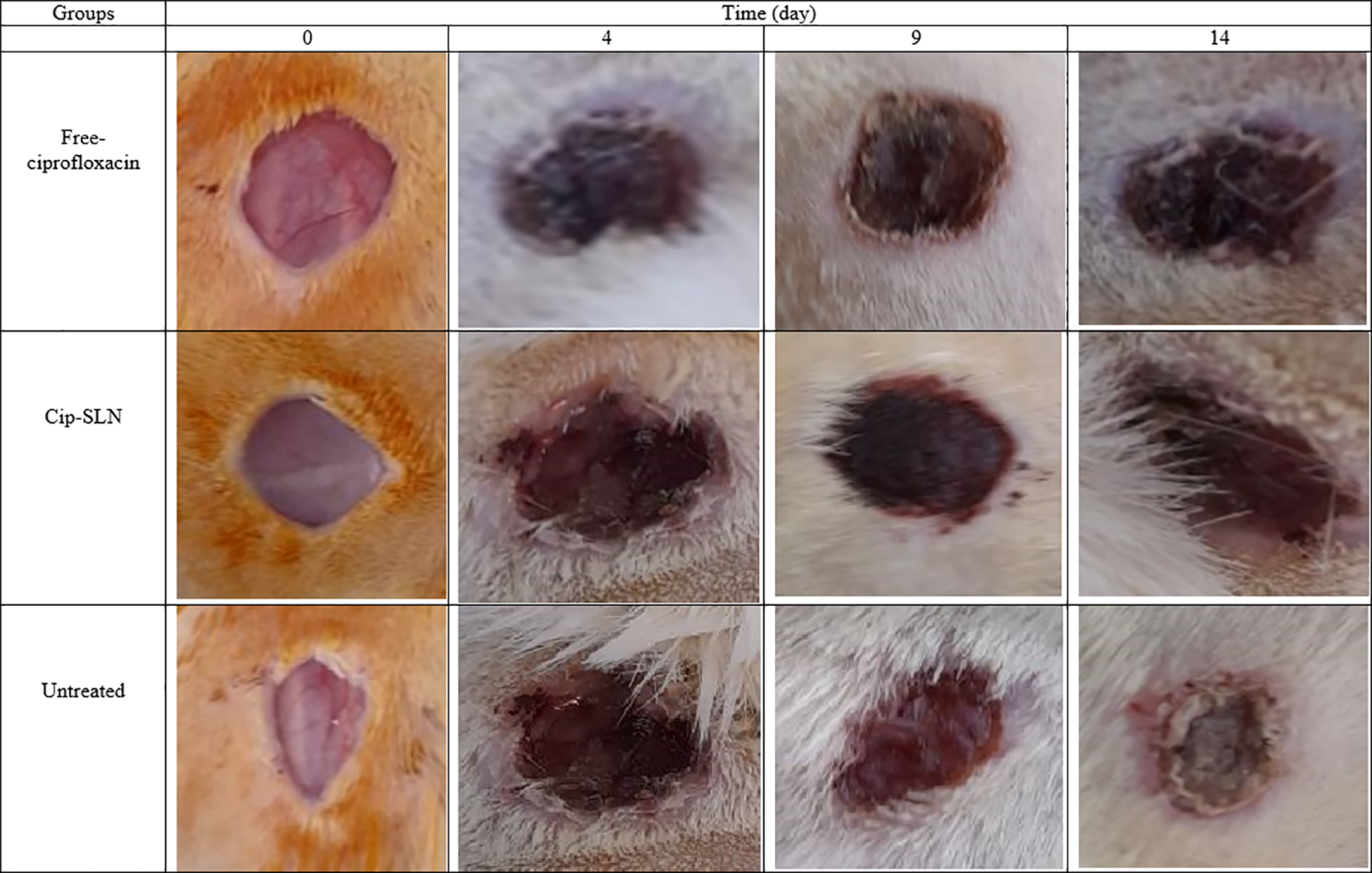
Representative images of change in skin and wound healing after treatment by free-Van-Amp and Van-Amp-SLN on days 0, 4, 9 and 14.

**Figure 8:**
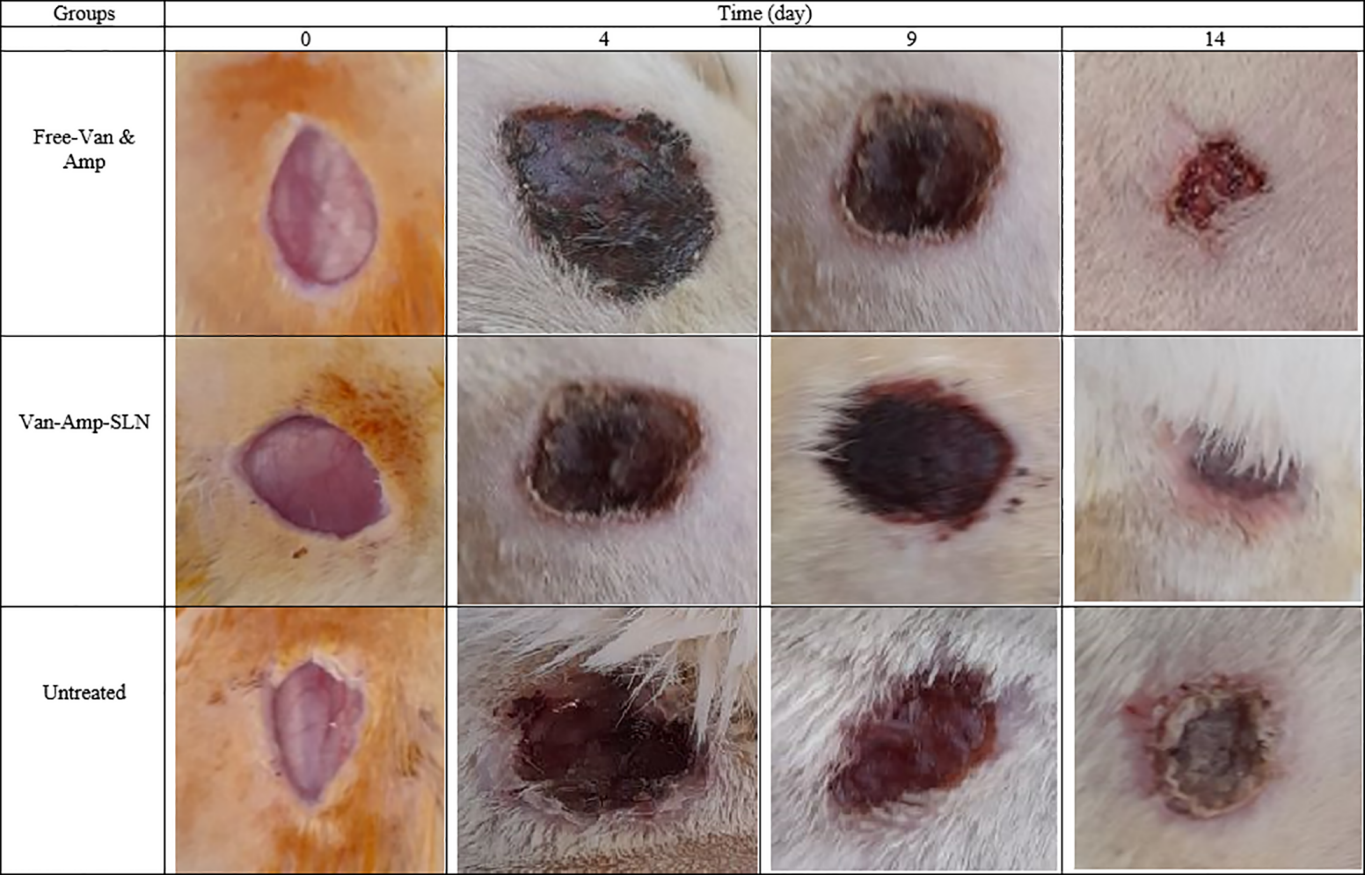
Representative images of change in skin and wound healing after treatment by free ciprofloxacin and Cip-SLN on days 0, 4, 9 and 14.

The wound healing process in the Amp-Van-SLN groups compared with the control group (untreated infection) during two weeks is shown in **Figure 7**. Due to the effect of Amp-Van-SLN on the ability to reduce the number of bacteria at the site of infection, wound healing occurred earlier. In general, after 2 weeks of wound formation, the wounds healed in all treatment and control groups. The wound healing process of groups receiving Cip-SLN on different days compared to the control group (infection without treatment) is shown in **Figure 8**. Because Cip-SLN can reduce the number of bacteria at the site of infection, wound healing occurs earlier. In general, after two weeks of wound formation, the healing process occurred in all treated and control groups.

### 3.6 Histological analysis

**Figure 9:**
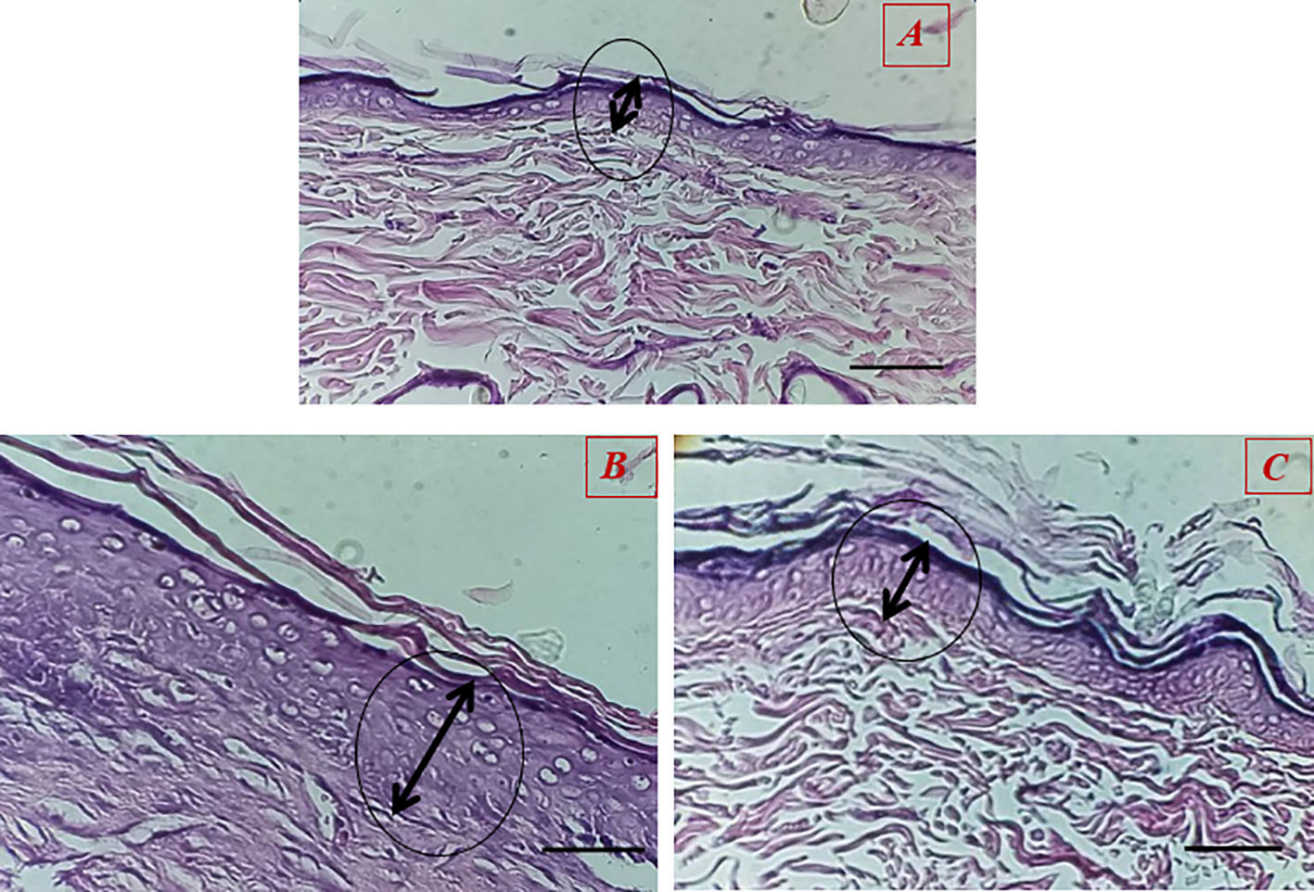
Histopathology of skin at day 14 stained with H&E, Epithelial layer thickness in treated groups **(A)** untreated groups, **(B)** Treated with Van-Amp-SLN, **(C)** Treated with free-Van-Amp.

**Figure 10:**
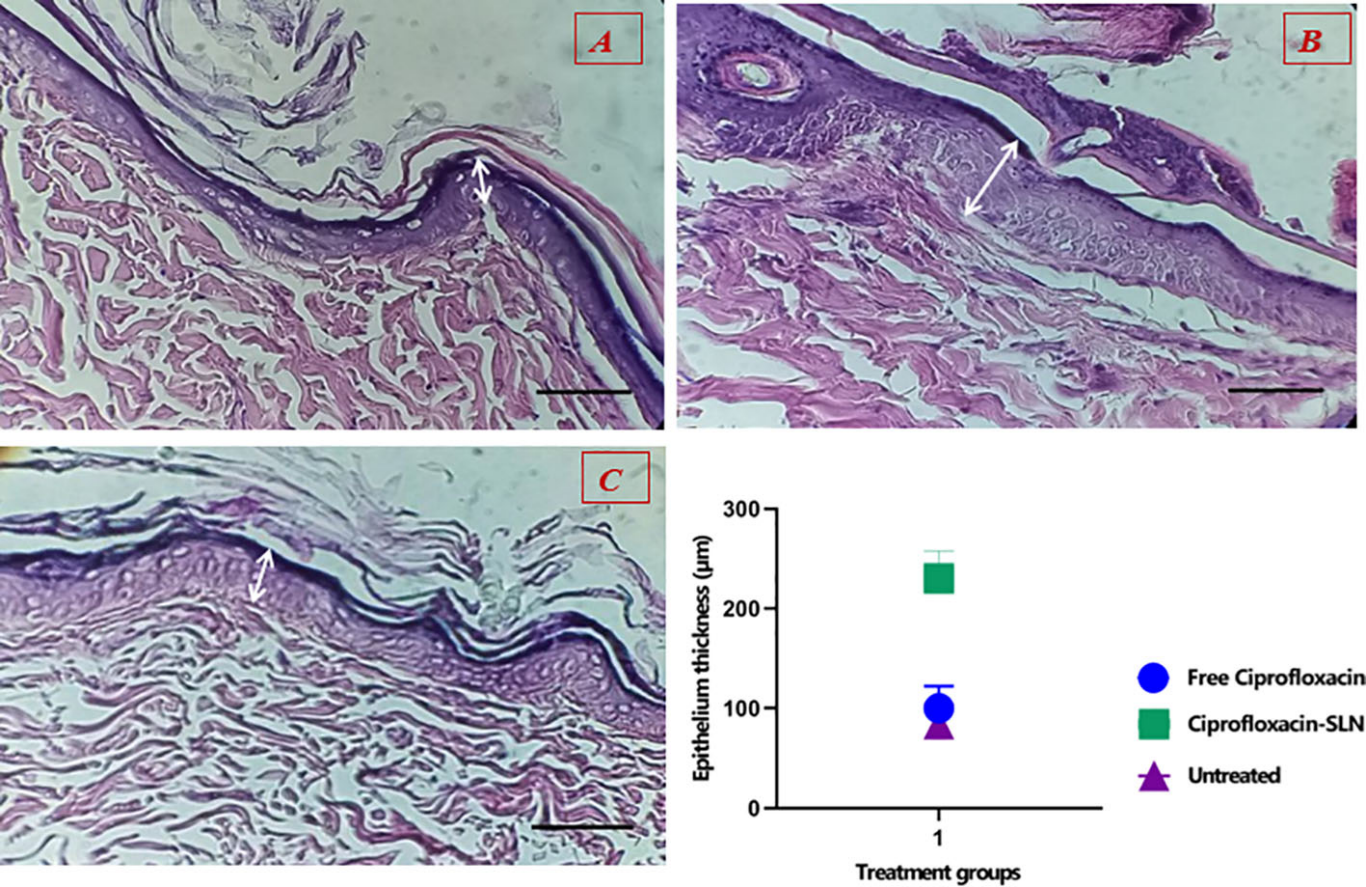
Histopathology of skin at day 14 stained with H&E, Epithelial layer thickness in treated groups **(A)** untreated groups, **(B)** Treated with Cip-SLN, **(C)** Treated with free ciprofloxacin.

The results showed that the healing process and regeneration of skin layers in all groups of rats were faster than in the control group. A comparison of the rate of epithelial cell formation in the treated groups is shown in **Figure 9**. It has been confirmed that using Van-Amp-SLN has about 50% greater epithelial diameter, confirming faster healing than other groups. **Figure 10** indicated that the use of Cip-SLN at the same time has 150% more epithelial diameter, which confirms faster recovery and healing than the group receiving the free drug.

## 4 Discussion

Antimicrobial resistance is a global health concern, and antibiotics have become ineffective against resistant infectious pathogens. MDR bacterial infections are estimated to kill 10 million people a year by 2050. Despite the availability of diverse antibiotic treatment options, their efficacy is constrained by the prevalence of drug-resistant bacteria. Moreover, this resistance contributes to economic repercussions, disease spread, and treatment failures, necessitating the development of innovative therapeutic approaches (19).

In this research, the double emulsion method was used to prepare nanoparticles which is a simple, inexpensive, and repeatable technique. This method needs excellent solvents and few amounts of surfactant. This method is a suitable structural model for encapsulating hydrophilic and lipophilic drugs such as ciprofloxacin, vancomycin, ampicillin, streptomycin, doxycycline, gentamicin and rifampicin (20–24). As the PDI value became closer to zero, the particles became more homogeneous. High zeta potential increases the stability of nanoparticles during treatment. Zeta potential is an important physical and chemical parameter affecting nanostructure stability (25). In the present study, the electric charge on the surface of the zeta potential particles was -18.5 ± 2.5 mV. This finding shows the high encapsulation efficiency without using organic solvents with less toxicity.

Stearic acid is a medium-chain fatty acid (molecular weight 284.48 g/mol) with a negative charge surface at neutral pH. Compared with the properties of any solid lipid, it is clear that stearic acid is the best choice for ciprofloxacin due to its good solubility. This lipid has a high drug-loading potential (26). In the optimal formulation, 600 mg of stearic acid was used, and the particle size was between 250 and 350 nm, but in the formulation that used 1200 mg, the particle size reached more than 400 nm.

Poloxamer 407 is a non-ionic surfactant that produces nanoparticles with a smaller size and less toxicity than Poloxamer 188, which prevents lipid matrix degradation *in vivo* (27). However, in this study, the use of Poloxamer 407 alone increased the size, PDI, and drug leakage. For this reason, a co-surfactant was used to solve this problem. Lipoid S-100 (derived from soy lecithin) was used in this study as a co-surfactant, which resulted in a smaller size, increased stability, and drug loading in the lipid matrix. Lipoid S-100 is a biodegradable and non-toxic phospholipid capable of emulsifying lipids and drugs to form water-in-oil or oil-in-water emulsions, which have been reported to be useful in various nanocarriers (27, 28). The present study confirmed that without the use of Lipoid S-100, the size of nanoparticles was higher than 700 nm, but with its use, the size was reduced to 250-350 nm.

According to the DSC results, it was determined that the drug is molecularly placed inside the lipid matrix. The disappearance of the peak related to ciprofloxacin in the DSC thermogram in the synthesized nanoparticle indicates the interaction of the drug with the lipid because the lipid tends to dissolve at 65°C. In the case of lyophilized nanoparticles, the peak shift of the drug to a lower temperature confirms its placement inside the solid lipid nanoparticles. One of the most important goals of the present study was the slow release of the drug from the nano-drug. The results showed that 82 hours are needed for 80% of the drug to be released from the synthesized nanoparticle. The nature of the lipid matrix, the concentration of surfactant and cosurfactant, and the parameters involved in the production of Cip-SLN are important and effective on the release of the drug from the nanoparticles, and the smaller the size of the nanoparticles, the duration of the release of the drug decreases due to the more contact of nanoparticles surface.

The findings of MIC and Well diffusion showed that there is no significant difference in the effect of free drug and nanoparticle on the studied bacterial strains, considering that in these methods, bacteria are directly exposed to the drug, so in the first 24 to 48 hours free form of antibiotics had a better effect than nanoparticles, but after 72 hours, the growth inhibition zone and the minimum inhibitory amount of nanoparticles and free form of antibiotics were not significantly different. It was similar to the results of the studies by Öztürk et al., which examined the properties of clarithromycin incorporated into SLN (29).

Another aim of this study was to investigate the effect of prepared nanoparticles on *P. aeruginosa* causing wound infection in laboratory animals. A comparison of the number of bacterial colonies in different treatment groups revealed that on the first day of the bacteria inoculation into the wounds, a large number of bacteria were seen at the site of infection. Sampling continued on days 4, 9, and 14. An important point and an interesting finding were that the rats that did not receive any drug, whether a free drug or nano-drug, recovered after 14 days and the number of bacteria in the wound site was significantly reduced. In the comparison between the drug-receiving groups and the untreated group, it was found that the bacterial colonies had a significant decrease (P<0.05). The results of comparing the treatment of infected wounds using nanoparticles and free drugs, it was found that nano-drugs have a better effect than free drugs and have reduced the number of bacteria at the site of infection. Cip-SLN had the best effect, which caused a 64% reduction of *P. aeruginosa* at the site of infection compared to the untreated group (P<0.05). The results of comparing the treatment of infectious wounds using Van-Amp-SLNs and free antibiotics showed that Van-Amp-SLNs have a better effect than free antibiotics and have reduced the number of bacteria at the site of infection. The best finding was related to Van-Amp-SLN, which reduced S. aureus by 66% at the site of infection compared to the untreated group (P <0.05).

These findings were consistent with the results of the study by Hajiahmadi et al. This group used liposomes loaded with vancomycin and lysostaphin to reduce the number of bacteria at the site of wounds infected with MRSA (17). Their findings showed that the use of liposomes loaded with vancomycin and lysostaphin significantly reduces the number of bacteria at the site of infection. One of the reasons for this is the slow and continuous release of the drug loaded in the nanoparticle matrix. Slow release results in the accumulation of more antibiotics at the infection site and may be more efficient than free medication. Wounds indicate a major health problem. Epithelialization, contraction, and deposition of connective tissue are processes that are effective in wound healing. The healing process largely depends on the regulated biosynthesis and deposition of new collagens and their subsequent maturation. In the process of tissue repair, inflammatory cells encourage the migration and proliferation of endothelial cells, leading to the neovascularization of connective tissue cells that synthesize extracellular matrices including collagen and keratinocytes, leading to re-epithelialization of the wounded tissue. Inflammation, collagen maturation, and scar formation are some of the multiple stages of wound healing that occur simultaneously but independently of each other (3, 17, 30).

Monitoring of the wounds infected by *P. aeruginosa* and different strains of *S. aureus* showed that after the injection of bacteria in the wound site, it takes 3 to 4 days for infected wounds to develop. The relative healing of the wound in all groups after two weeks of wound formation indicates wound healing in healthy rats. However, an important point was the healing time of wounds in different treated groups. The results showed that the lower number of bacteria in the wound could help the faster healing process. As seen in **Figures 7** and **8**, the wounds of rats treated with nanoparticles were cured 5 days earlier. Jun Tian and colleagues used silver nanoparticles to cure burns in rats and their findings showed that the use of silver nanoparticles caused burn healing 10 days earlier compared to free drugs and routine drugs used for burns (31).

Nikpasand et al. used titanium dioxide nanoparticles/gelatin to heal infected skin wounds and determined by daily measurement of the diameter of the created wounds that the use of these nanoparticles leads to faster healing of the wounds (32). This finding is consistent with the results of the present study. According to the results of pathology studies, it was found that the process of making epithelium happens faster in treated rats compared to untreated ones. According to **Figures 9** and **10**, the diameter of the epithelium formed in the group of rats receiving nanoparticles is significantly increased compared to rats receiving free drugs.

Xiaoxiao Wen et al. used silver sulfadiazine nanoparticles to treat burn infection wounds caused by *E.coli* and *P. aeruginosa*, their results showed that the use of nanoparticles has a greater effect on inhibiting bacteria and also causes faster healing of the wound, which is confirmed by our study (33). Other studies have also used nanoparticles to treat wound infections (34–37). However, the use of SLNs showed promising results in wound healing due to their high persistence on the wound surface. In the future, it is suggested to conduct studies using antibiotics or natural compounds such as curcumin using solid lipid nanoparticles in the treatment of infected wounds.

## 5 Conclusion

The results of this study revealed that by reducing the number of bacteria in infected wounds, the wound healing process happens faster. The use of SLNs due to their fatty nature and sufficient drug loading can increase the number of antibiotics at the site of infection with slow drug release, which leads to greater effects on reducing the number of bacteria and faster wound healing.

## Data Availability

The datasets presented in this study can be found in online repositories. The names of the repository/repositories and accession number(s) can be found in the article/supplementary material.
